# The Value of LncRNA BCAR4 as a Prognostic Biomarker on Clinical Outcomes in Human Cancers

**DOI:** 10.7150/jca.35113

**Published:** 2019-10-15

**Authors:** Chao Tu, Xiaolei Ren, Jieyu He, Chenghao Zhang, Ruiqi Chen, Wanchun Wang, Zhihong Li

**Affiliations:** 1 Department of Orthopedics, The Second Xiangya Hospital, Central South University, Changsha, Hunan, P.R. China.; 2 Hunan Key Laboratory of Tumor Models and Individualized Medicine, The Second Xiangya Hospital, Central South University, Changsha, Hunan, P.R. China.; 3 Department of Geriatrics, The Second Xiangya Hospital, Central South University, Changsha, Hunan, P.R. China.; 4 University of Texas Health Science Center at San Antonio, San Antonio, Texas, USA.

**Keywords:** LncRNA, BCAR4, cancer, sarcoma, prognosis, metastasis

## Abstract

**Background**: This updated meta-analysis aimed to analyze available data to explore the prognostic value of long noncoding RNA breast cancer anti-estrogen resistance 4 (BCAR4) in various human malignancies. **Methods**: Literature retrieval was performed by systematic searching several authoritative databases, including Pubmed, PMC database, Web of Science, the Cochrane Library, Embase, and CNKI database up to Feb 10, 2019. Data were extracted and subsequently crosschecked, and discrepancies were discussed to reach consensus. Quality of the eligible studies was evaluated by Newcastle-Ottawa Scale (NOS). The fixed- or random-effects model was used to calculate the pooled the hazard ratios (HRs) or odds ratios (ORs) and the 95% confidence interval (95% CI). Publication bias was detected by using Begg's funnel plot and Egger's test. **Results**: A total 1,128 cancer patients from thirteen studies were included and pooled in the present meta-analysis. High expression levels of BCAR4 were correlated with unfavorable overall survival (OS) (HR=2.23, 95% CI: 1.84-2.71), but not progression-free survival (PFS) (HR=1.30, 95% CI: 0.80-2.11). Subgroup stratified analysis showed that tumor type, sample size, follow-up months, and survival analysis method did not alter the predictive value of BCAR4 on OS in various cancers. Furthermore, elevated BCAR4 level was markedly correlated with advanced clinical stage (III/IV) (OR=3.28, 95% CI: 2.33-4.60), and dramatically predicted lymph node metastasis (OR=3.00, 95% CI: 1.95-4.63, *P*<0.00001) and distant metastasis (OR=3.36, 95% CI: 1.88-5.98, *P*<0.0001), but not associated with age, gender or tumor size. No obvious heterogeneity was noted for correlation between BCAR4 expression and OS across these studies. **Conclusions**: High expression of BCAR4 was correlated with unfavorable overall survival outcome and clinical features including metastasis and progression, implicating an independent prognostic value for BCAR4 in human cancers.

## Introduction

Cancer is predicted to rank as the leading cause of mortality and the single most critical barrier to increase life expectancy worldwide over the past decades 1. An estimated 18.1 million new cancer cases and 9.6 million cancer deaths worldwide in 2018 were reported by the International Agency for Research on Cancer 1. Despite tremendous achievements have been made in surgery, adjuvant radio- and chemotherapy, targeted therapy 2, and even immunotherapy 3 in the past decades, the prognosis and quality of life of cancer patients still remain poor, largely due to the shortage of effective and noninvasive predictive factors during early stage of malignancies. Therefore, many researchers have been devoted to exploration of new promising putative biomarkers for prognosis and therapeutic efficacy for cancer patients, and finally improve their survival outcomes 4.

Non-coding RNAs (ncRNAs) refer to a variety type of RNA with a profound role in epigenetic regulation at the transcriptional and post-transcriptional level, including hetero-chromatin formation, DNA methylation, gene silencing, and histone modification 5-7. Generally, the epigenetic related ncRNAs can be simply separated into two main categories by their size: those with nucleotides less than 30 nts are short ncRNAs, such as microRNAs (miRNAs), piwi-interacting RNAs (piRNAs), and short interfering RNAs (siRNAs), while those longer than 200 nts belong to long ncRNAs (lncRNAs) 8-10.

LncRNAs could drive many pathophysiologic phenotypes through their interaction with other cellular macro-molecules including DNA, RNA and proteins 11. Accumulating evidences have linked expression or functional abnormalities of lncRNA with various complex human disease, such as aging 12, degenerative disease 13, and coronary artery disease 14. Recently, lncRNAs have been reported to function in biological processes associated with cancer initiation and progress including proliferation, apoptosis and invasion, and therefore implicate a putative role in tumorigenesis 7, 15-17.

Breast cancer anti-estrogen resistance 4 (BCAR4) gene produces a spliced lncRNA that has been firstly identified to be inversely associated with the development of resistance to anti-estrogens in breast cancer cells and poor disease-free survival (DFS) for recurrent breast cancer. Previously, lncRNA BCAR4 was considered as an oncogene and was reported to play a pivotal role in metastasis and tamoxifen-resistance of breast cancer 18. Recently, many researches showed that the dramatically elevated expression pattern of BCAR4 was closely correlated with worse survival and high risk of metastasis in other cancer patients as well 19. The expression of BCAR4 was higher in various tumor tissues than normal tissue or para-tumor tissue, including breast cancer 20-22, non-small-cell lung cancer 23-25, prostate cancer 26, osteosarcoma 27, 28, gastric cancer 29, cervical cancer 30 and colorectal cancer 31, 32. However, most individual studies evaluating BCAR4 expression in cancers remain insufficient due to the limitations in small sample size and possible controversial outcomes. Accordingly, we conducted this comprehensive meta-analysis with all related eligible studies and pooled results to further address the feasibility of BCAR4 as a noninvasive prognostic biomarker candidate.

## Material and methods

### Search strategy and literature selection

Potential eligible literature that related to the prognosis and metastasis of BCAR4 and human cancer were thoroughly searched in related databases, including Pubmed, PMC database, Web of Science, the Cochrane Library and Embase, as well as Chinese databases: China National Knowledge Infrastructure (CNKI) from inception to Feb 10, 2019. The searched terms in variably combinations were listed as follows: (“long noncoding RNA-, lncRNA-, breast cancer anti-estrogen resistance 4, BCAR4,”) and (“carcinoma” or “sarcoma” or “cancer” or “tumor” or “tumour” or “neoplasm” or “malignancy”). An additional manual search of references lists of primary literature was performed to find supplementary pertinent articles. Notably, the current study was critically projected, reviewed and reported in accordance with the PRISMA checklist to enhance the credibility of the results 33, 34.

### Inclusion and exclusion criteria

Rigorous inclusion and exclusion criteria were adopted in this study. Inclusion criteria were as following: 1) articles examining the clinical prognostic value of BCAR4 in any malignancies; 2) the patients had been grouped according to the BCAR4 expression (high versus low); 3) definite diagnosis with histopathology confirmation; 4) Adequate data for the measurement of hazard ratios (HRs) or odds ratios (ORs) and the corresponding 95% confidence intervals (CIs); and 5) published in English or Chinese language.

By contrast, exclusion criteria were as following: 1) literature not pertinent to the BCAR4; 2) studies concerning the structure or functions of BCAR4; 3) multiple duplicate publications or duplicate data in the different works, excluding smaller sample data; 4) animal experiments, and 5) absence of usable clinical data or documents without original data, such as correspondences, editorial materials, case reports or reviews.

### Data extraction and quality assessment

Two investigators (CT and XLR) extracted all the essential information from identical articles independently, and a third investigator (JYH) was consulted to reach a consensus when inconsistencies exist between the investigators. Extracted information are as following: 1) first author' name, year of publication, origin country, tumor type, sample size, follow-up months, cut-off value, clinical TNM stage, detection and survival analysis method; 2) HRs or ORs with 95% CI of BCAR4 for overall survival (OS), progression-free survival (PFS), DFS, recurrence-free survival (RFS), lymph node metastasis (LNM) and distant metastasis (DM). If only Kaplan-Meier curves were provided in certain studies, the survival rates were indirectly extracted from the graphical plots and calculated HRs with 95% CIs were determined by using Engauge Digitizer software (Version 4.1) as previously described 35. Moreover, best efforts were made by contacting the corresponding author to obtaining the possible data if they were not available from the enrolled articles.

Quality assessment of the eligible literature was performed by two independent investigators (CHZ and RQC) through using the Newcastle-Ottawa Scale (NOS), and the studies with NOS score ≥7 were considered to be of high quality 36.

### Data synthesis and statistical analysis

All statistical analyses were conducted on STATA software (Version 12.0) and Review Manager (RevMan 5.3). The impact of BCAR4 expression on clinical characteristics, prognosis and metastasis was described as HRs or ORs with corresponding 95% CIs. Heterogeneity among the included studies was quantified with the by chi-squared test and *I^2^* statistics. A chi-squared test of *p*<0.10, *I^2^*>50% indicated significant heterogeneity across the studies, and the random-effects model should be adopted in analyzing the pooled results. On the contrary, the fixed-effects model could be applied in data analysis when no obvious heterogeneity was detected (chi-squared test of *p*>0.10 and *I^2^*<50%). Probable publication bias was estimated by employing Begg's funnel plot as well as Egger's regression test. *P*<0.05 of the two-tailed probability was considered to be statistically significant.

### Verification of results from TCGA and GTEx dataset

Gene Expression Profiling Interactive Analysis (GEPIA) was additionally used in this meta-analysis in order to further verify the expression levels of BCAR4 in cancerous and normal tissue and its correlations with OS and DFS from The Cancer Genome Atlas (TCGA) and GTEx dataset. The survival analysis was calculated by Kaplan-Meier (K-M) method and logrank test, and the HRs and p-value were shown in the figure of K-M curves as previously described 37.

## Results

### Included literature

**Figure 1:**
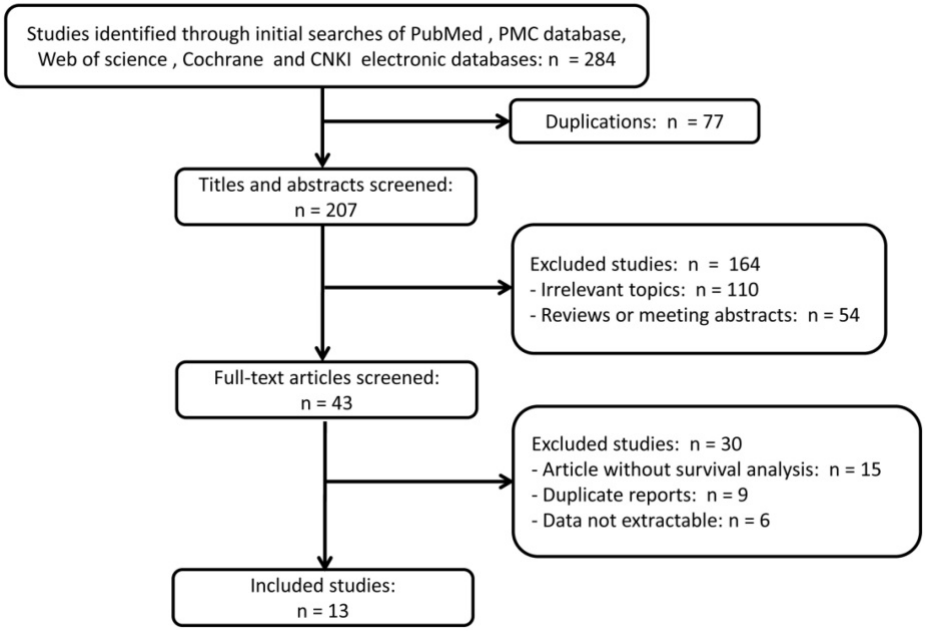
Flow diagram of study identification with criteria in the meta-analysis.

A total of 284 references were retrieved through initial searches of the electronic databases above-mentioned. 77 duplication articles were excluded firstly after screening. 164 articles, including 110 studies on irrelevant topics and 54 reviews or meeting abstracts, were further excluded according to the inclusion and exclusion criteria after examination by title and abstract. For the remaining 43 potential candidate studies, full texts were further carefully reviewed, and 15 articles were excluded as survival analysis was not described, nine studies were duplicate reports from the same research organizations, and six are abstracts which data are not extractable. Ultimately, thirteen articles were included and used in quantitative synthesis for the present systematic review and meta-analysis. The selection process was briefly presented in the flow diagram in Figure 1.

### Characteristics of the enrolled studies

The main features of the thirteen included articles are concisely summarized in Table 1. These studies were published between 2010 and 2018 with sample size ranging from 30 to 168. The median or mean value was selected as the cut-off value in most articles. Eleven of the studies were obtained from China, whereas one from the US and another from the Netherlands. Out of the thirteen studies, three emphasized breast cancer, while another was based on castration-resistant prostate cancer. Additionally, two of them focused on osteosarcoma, with another three focusing on non-small cell lung cancer. There were also two based on colorectal cancer, another one taking into gastric cancer, and the last one emphasized cervical cancer. All of the 1,128 patients were divided into two distinct groups (high and low expression of BCAR4) as measured by qRT-PCR or RNA *in situ* hybridization. Ten of thirteen studies investigated the association between BCAR4 expression and OS, while only two depicted BCAR4's prognostic role in PFS.

The NOS score indicated the overall good quality of the studies (median, 7.8 points; range, 7-9), with no manuscripts displaying high risk of bias.

### BCAR4 and main survival outcome

**Figure 2:**
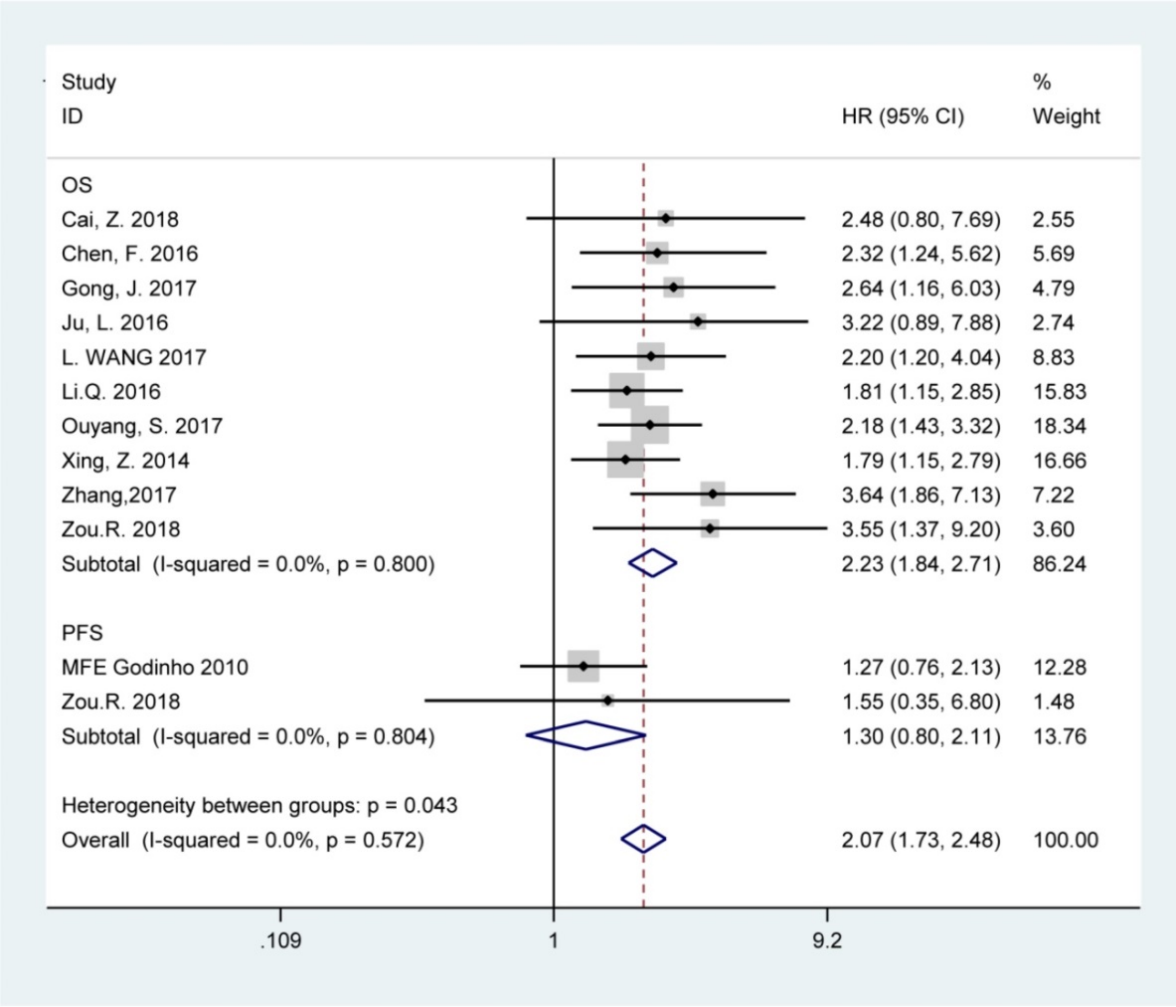
Forest plots for the association between BCAR4 expressions with overall survival (OS) and progression-free survival (PFS).

The fixed-effects model was used to analyze the pooled HRs and corresponding 95% CIs since no obvious heterogeneity was noted among the studies involved in OS and PFS analysis (chi-squared test of *p*>0.10 and *I^2^*<50%). Elevated BCAR4 expression was predictive of unfavorable OS (HR=2.23, 95% CI: 1.84-2.71), but not PFS (HR=1.30, 95% CI: 0.80-2.11) in various carcinomas according to the results (Figure 2).

**Figure 3:**
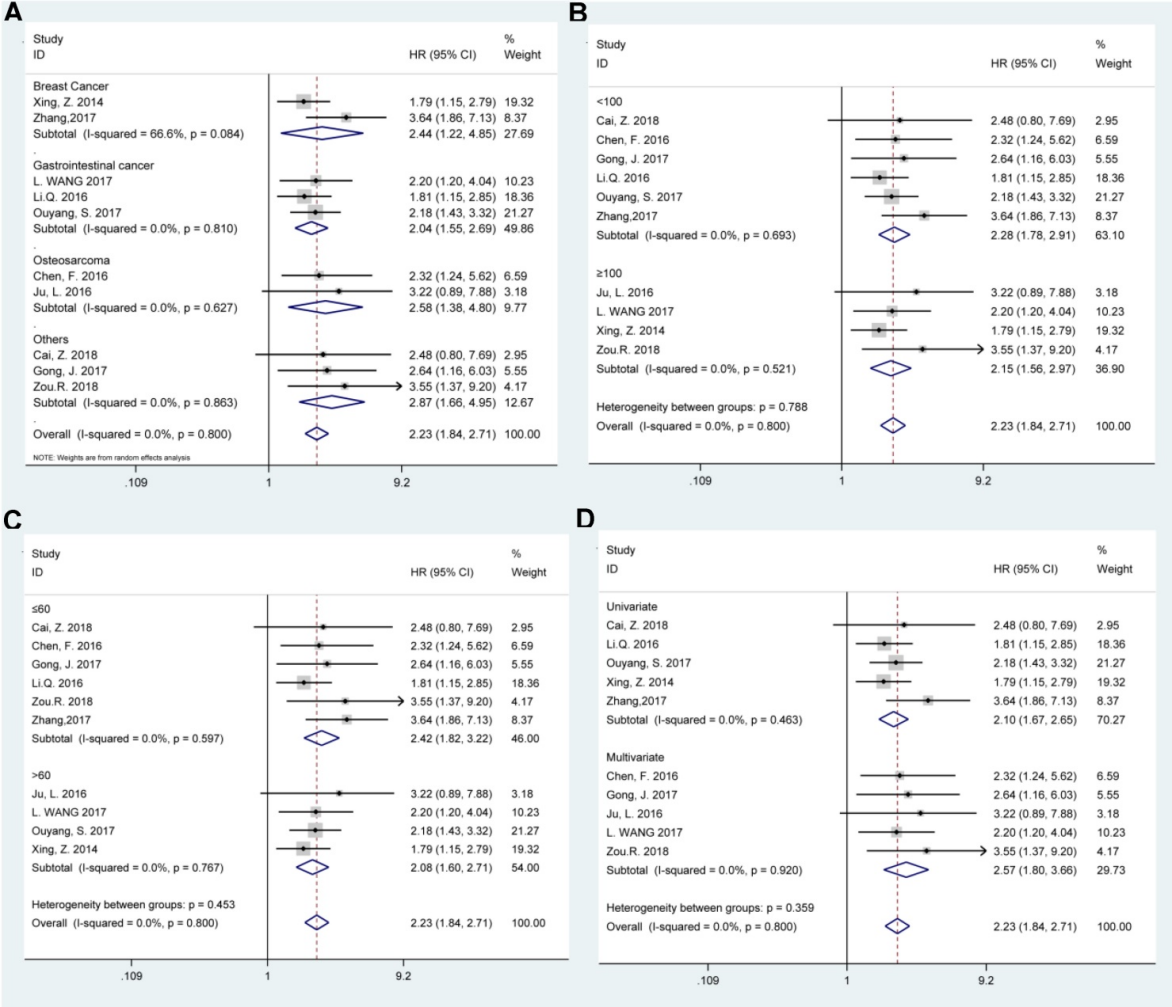
Stratified analyses for the correlation between BCAR4 expressions with overall survival (OS). Subgroup analysis of pooled HRs of OS by factor of tumor type (A), sample size (B), follow-up months (C), and survival analysis method (D) were presented respectively.

Afterwards the stratified analyses were conducted by tumor type, sample size (more or less than 100), follow-up months (more or less than 60), and survival analysis method to further analyze the BCAR4 expression with OS, as displayed in Table 2. The results showed that all these subgroup analysis parameters did not alter the prognostic value of BCAR4 on OS (Figure 3). Of note, for studies assessing OS in different tumor types, the results indicated that promoted BCAR4 levels could significantly predict worse outcome in breast cancer, gastrointestinal cancer, and osteosarcoma with pooled HRs with 95% CIs of 2.44 (1.22, 4.85), 2.04 (1.55, 2.69) and 2.58 (1.38, 4.80) respectively as shown in Figure 3.

### BCAR4 and other clinicopathological features

The characteristics of the enrolled studies which assessing the correlation between BCAR4 expression and other clinicopathological features including metastasis were summarized in Table 3.

**Figure 4:**
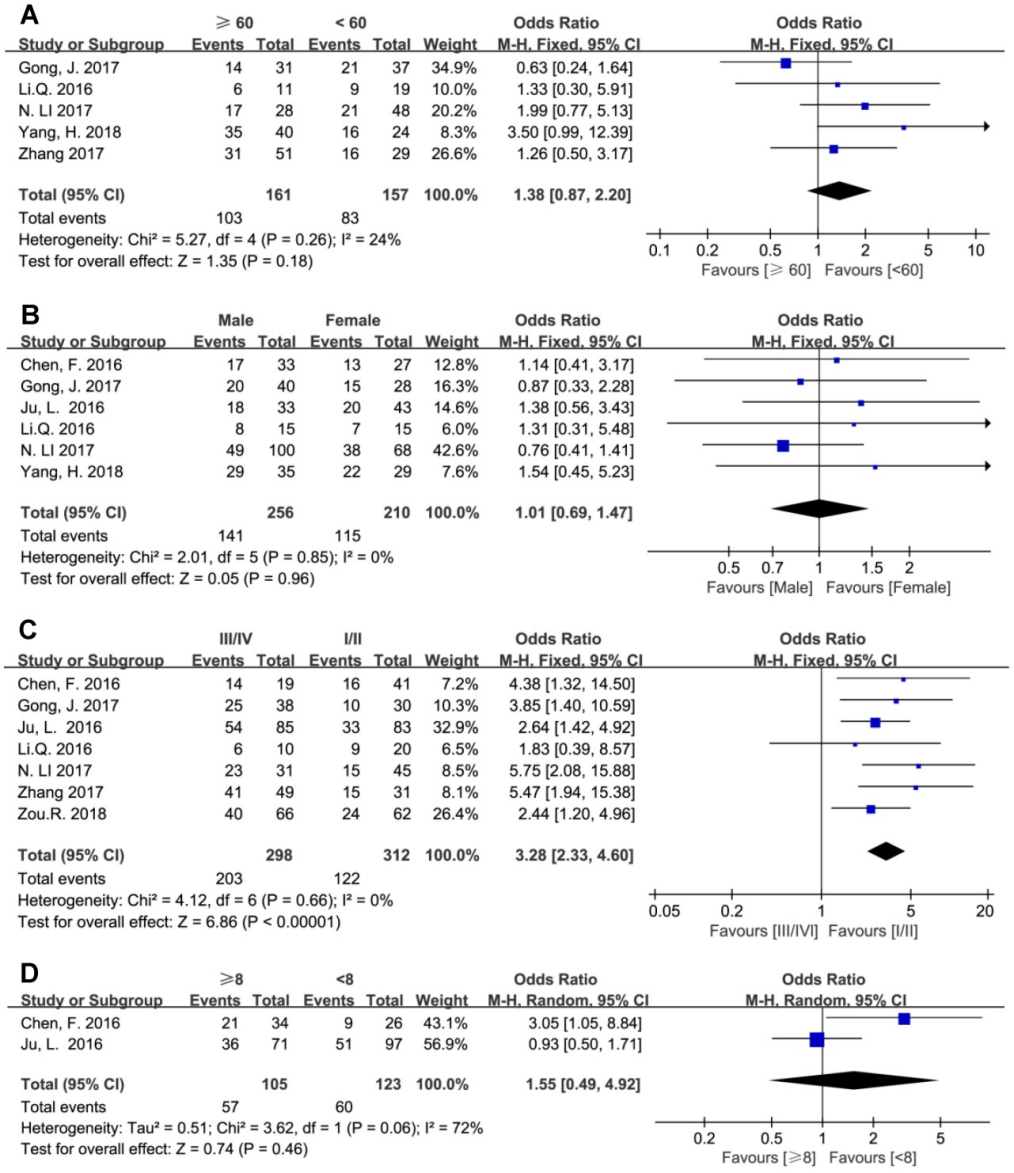
Forest plots for the association between BCAR4 expressions with other clinicalpathologic features, including age (A), gender (B), clinical stage (C) and tumor size (D).

The results showed that elevated BCAR4 expression was positively associated with advanced clinical TNM stage (III/IV vs. I/II) with estimated OR and 95% CI of 3.28 (2.33, 4.60), while age, gender and tumor size showed no correlation with BCAR4 level (Figure 4).

**Figure 5:**
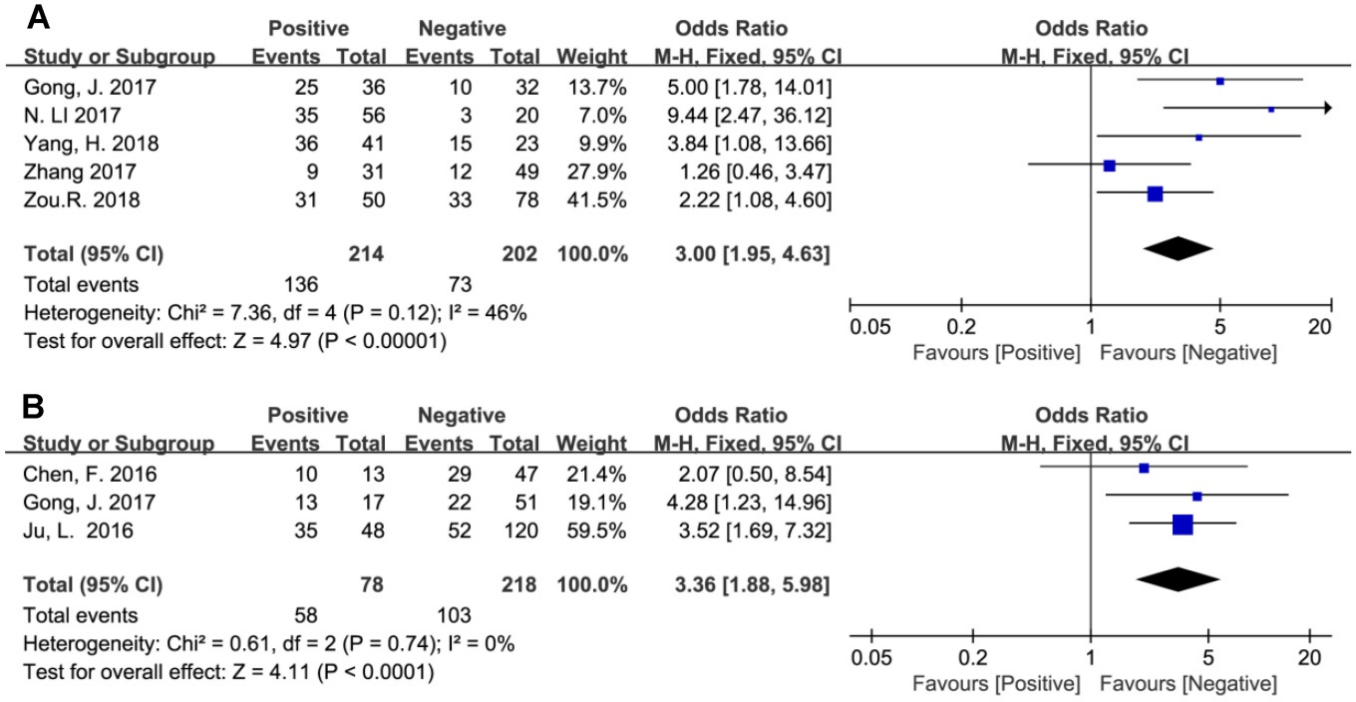
Forest plots for the association between BCAR4 expression with lymph node metastasis (A) and distant metastasis (B).

In addition, the pooled ORs have revealed that BCAR4 expression might be regarded as an independent prognostic biomarker for aggressiveness and metastasis in human cancers. As presented in Figure 5, promoted BCAR4 expression strongly predicted LNM (OR=3.00, 95% CI: 1.95-4.63, *P*<0.00001) and DM (OR=3.36, 95% CI: 1.88-5.98, *P*<0.0001) respectively.

### Sensitivity analysis and publication bias

**Figure 6:**
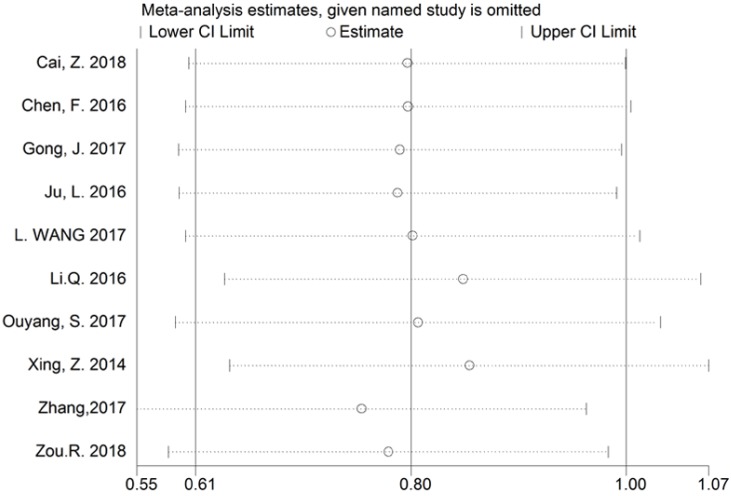
Sensitivity analysis of BCAR4 expression for overall survival (OS).

The reliability of the crude results was evaluated by sensitivity analysis. After exclusion of any individual study, the combined effect of the pooled HR revealed no significant change, and therefore the results regarding BCAR4 expression for OS are considered to be credible (Figure 6).

**Figure 7:**
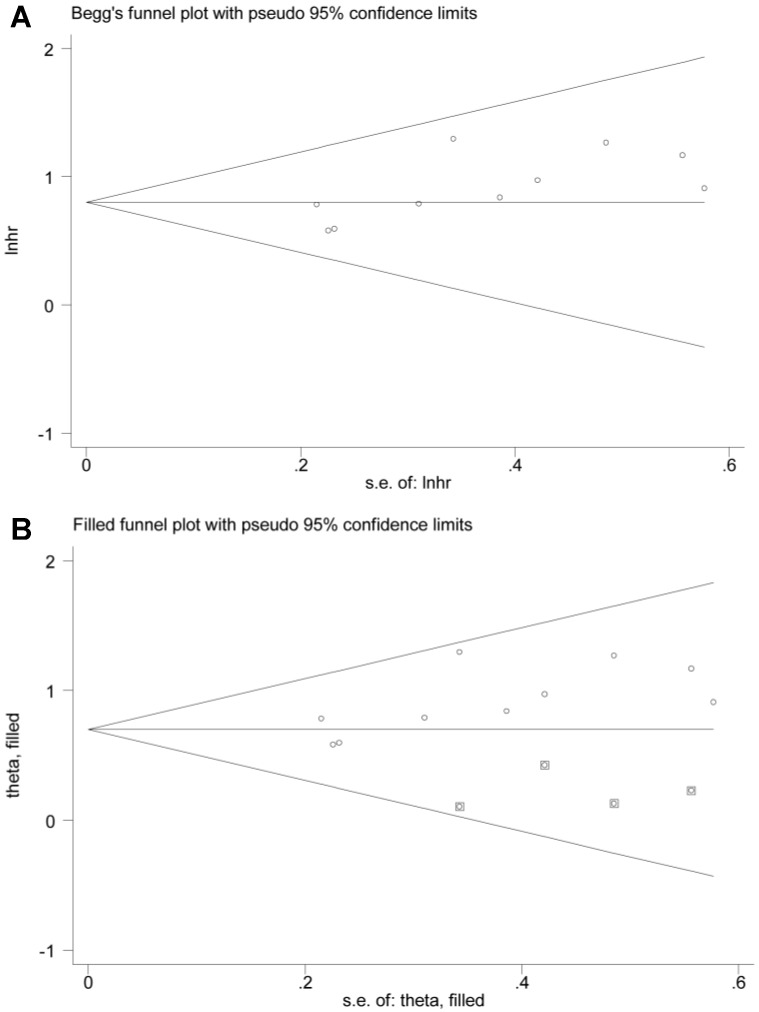
Publication bias of BCAR4 expression for overall survival (OS): Begg's funnel plot (A) and filled funnel plot (B) after adjustment by using the “trim-and-fill” method.

The potential publication bias of the present meta-analysis was assessed by Begg's funnel plot, as well as Egger's test. However, the Egger's regression test indicated significant bias, and the shape of Begg's funnel plot also revealed evidence of asymmetry (Figure 7A). Therefore, we performed nonparametric “trim-and-fill” method by adding four missing studies as previously described (Figure 7B) 38. The pooled HR and corresponding 95% CI for BCAR4 expression on OS was 2.01 (1.69-2.40) after adjustment.

### Different BCAR4 expression levels in cancer from TCGA and GTEx database

In addition, we compared BCAR4 expression levels between cancerous and normal tissues in patients with different kinds of cancers using TCGA and GTEx datasets to verify the expression status or levels of lncRNA BCAR4. A majority of cancers showed higher expression of lncRNA BCAR4 in tumor tissues when compared with normal tissues, including sarcoma (SARC), breast invasive carcinoma (BRCA), cervical squamous cell carcinoma and endocervical adenocarcinoma (CESC), lung adenocarcinoma (LUAD), lung squamous cell carcinoma (LUSC), prostate adenocarcinoma (PRAD), and stomach adenocarcinoma (STAD). The details are showed in Figure S1.

## Discussions

LncRNAs are non-protein-coding transcripts that were previously defined as chunk RNA and transcriptional “noise” 39. However, this perception has been steadily replaced in the past few years since recent advancements in surveying mechanisms of lncRNAs have provided tools to functionally annotate these transcripts in diverse cellular processes 11, 40. With rapid development of next-generation sequencing technique, mounting researches have uncovered the role of lncRNAs in regulating target gene expression as oncogenic or tumor suppressors 41. Consequently, lncRNAs have been proposed as promising biomarkers for early detection and accurate prognosis for various neoplasms nowadays 42-44.

Recent studies have investigated the association between lncRNA BCAR4 and human cancers, and the results show that high expression of BCAR4 indicates aggressiveness and poor prognosis in various carcinomas 19, including osteosarcoma 27, 28, breast cancer 20, 21, 45, 46, non-small cell lung cancer (NSCLC) 23, 25, gastric cancer 29, prostate cancer 26, colorectal cancer 31, 32, 47, and cervical cancer 30. Moreover, higher expression levels of BCAR4 in most cancer tissues compared with normal tissues were verified using TCGA and GTEx databases. However, results from these studies should be interpreted with caution due to the limited sample size and discrete outcomes. A meta-analysis has been conducted by Zhao W. and colleagues 19 to demonstrate the pooled prognostic value of BCAR4 in human cancers. However, only nine studies were included without extensively searching other databases or providing information regarding BCAR4 and other clinical parameters, such as PFS, tumor size, gender and age. Of note, one study performed by MFE Godinho 20 does not contain data regarding the comparison of OS between high and low expression levels of BCAR4, whose inclusion in the meta-analysis might introduce possible bias to Zhao's results 19. Therefore, we conducted this updated comprehensive meta-analysis to further investigate the prognostic role of BCAR4 in various cancers. Thirteen studies with seven cancer types containing 1,128 patients were pooled together in this study, and the results indicate that promoted BCAR4 expression was markedly associated with poor prognosis of OS, but not PFS in patients with a variety of cancers. Furthermore, subgroup stratified analysis showed that tumor type, sample size, and follow-up months, and survival analysis method did not alter the predictive value of BCAR4 on OS in human cancers. In addition, elevated BCAR4 level was markedly correlated with advanced (III/IV) clinical TNM stage, and dramatically predicted LNM and DM. However, the included single cohort studies showed that there were no significant difference between BCAR4 expression and TNM stage, LNM and DM, which were discordant with the pooled results due to the limited sample size 27, 31. Besides, the pooled OR implied BCAR4 expression levels were not associated with age, gender or tumor size. Despite a mild publication bias regarding BCAR4 expression for OS was observed in the study, the adjusted estimated value (HR=2.01, 95% CI: 1.69-2.40) was not significantly different from the previous data (HR=2.23, 95% CI: 1.84-2.71) after using the “trim-and-fill” method, indicating the credibility of our results.

As one of the promising prognostic biomarkers with high accuracy for various patients, BCAR4 has also been claimed to be involved in diverse biological processes in cancers 48. For instance, BCAR4 could activate mTOR pathway to induce cell proliferation and migration in chondrosarcoma 49, and regulate the expression of β-catenin by Wnt signaling pathway to promote the drug-resistance in gastric and breast cancer 20, 29. Besides, BCAR4 could wire up the Hippo pathway effector- Yes-associated protein (YAP) and Hedgehog (Hh) signaling to reprogramme glucose metabolism in breast cancer 18. Co-expression of BCAR4 and low level of *ERBB2* occurs frequently and indicate a worse PFS outcome for breast cancer patients undergoing tamoxifen resistance 45. Moreover, the overexpression of BCAR4 could upregulate glioma-associated oncogene 2 (*GLI2)* level and promote cancer cell viability, migration and invasion both *in vitro* and *in vivo* 24.

Whereas BCAR4 knockdown could significantly suppress tumor cell proliferation, invasion and metastasis, as well as induce cell cycle arrest and increase apoptosis in NSCLC and cervical cancer 25, 50. In addition, BCAR4 could mediate either canonical or non-canonical Hh cascade to activate *GLI2*-dependent gene transcription 30, 48, or regulate epithelial-mesenchymal transition (EMT) 49, and subsequently promote cell growth, metastasis and invasion in breast cancer and non-small cell lung cancer, or contribute to castration resistance in prostate cancer 26.

Taken together, the results of our comprehensive meta-analysis have demonstrated that BCAR4 expression is strong associated with unfavorable OS outcome and aggressive clinical features including metastasis and progression, suggesting an independent prognostic value for BCAR4 in human cancers and providing some insights for further research. However, it should be noted that several limitations still remain. First, some of the HRs were calculated by reconstructing survival curves rather than directly obtained from the original data, which might induce inevitable bias. Second, the cutoff value for BCAR4 expression varied across different studies due to the difficulty in reaching a consensus value, thus may introduce possible bias to the meta-analysis. Third, a majority of patients enrolled in our study were from China, except for one from the US and another from the Netherlands. Since discrepancy may exist among different races, our results may not be able to generalize to a larger spectrum of patients in other ethnicities and regions. Fourth, sample size of these studies is still small. Well-designed cohort studies with a larger sample size need to be carried out to further validate our results. Finally, the underlying mechanisms of BCAR4 in cancer progression still remain poorly understood, and thus more effort should be expanded to thoroughly elucidate the causative link between BCAR4 and human cancers.

In conclusion, the results of our study demonstrated strong correlation of BCAR4 with unfavorable survival outcome and clinical features including metastasis and progression, implicating an independent prognostic value for BCAR4 in human cancers. However, it should be noted that well-designed clinical studies with larger sample size are still warranted to clarify the predictive role of BCAR4 in cancer prognosis in future.

## Supplementary Material

Supplementary figures.

## Figures and Tables

**Table 1:** Summary of the main characteristics of the included studies.

First Author	Year	Country	Tumor Type	TNM Stage	Sample Size	BCAR4 expression		Cutoff Value	Follow-up (months)	Detection Method	Survival Analysis	Outcome Measure	NOS
						High	Low						
Cai, Z 26	2018	China	PC	II-IV	40	N/A	N/A	Median	60	qRT-PCR	Univariate	OS	7
Chen, F 27	2016	China	Osteosarcoma	I-IV	60	30	30	Median	60	qRT-PCR	Multivariate	OS/RFS	7
Gong, J 23	2017	China	NSCLC	I-IV	68	35	33	Mean	60	qRT-PCR	Multivariate	OS	8
Ju, L 28	2016	China	Osteosarcoma	IIA-III	168	87	81	N/A	70	qRT-PCR	Multivariate	OS	8
L. WANG 29	2017	China	GC	N/A	113	N/A	N/A	Mean	150	qRT-PCR	Multivariate	OS	9
Li, Q 31	2016	China	CRC	I-IV	30	15	15	N/A	30	qRT-PCR	Univariate	OS	7
MFE Godinho 20	2010	The Netherlands	BC	N/A	81	40	41	Median	120	qRT-PCR	Univariate	PFS/MFS	9
N. Li 25	2017	China	NSCLC	I-IV	76	38	38	N/A	N/A	qRT-PCR	N/A	None	7
Ouyang, S 32	2017	China	CRC	I-III	60	N/A	N/A	N/A	90	RNA Hybridization	Univariate	OS/DFS	8
Xing, Z 21	2014	The US	BC	N/A	160	N/A	N/A	N/A	150	qRT-PCR	Univariate	OS	7
Yang, H 24	2018	China	NSCLC	N/A	64	51	13	N/A	64	qRT-PCR	Univariate	None	7
Zhang, JB 22	2017	China	BC	I-IV	80	47	33	N/A	<60	qRT-PCR	Univariate	OS	8
Zou, R 30	2018	China	Cervical cancer	I-IV	128	64	64	Median	60	qRT-PCR	Multivariate	OS/PFS	9

Abbreviations: BC, breast cancer; CRC, colorectal cancer; DFS, disease-free survival; GC, gastric cancer; MFS, metastasis-free survival; N/A, not available; NSCLC, non-small cell lung cancer; OS, overall survival; PC, Prostate cancer; PFS, progression-free survival; RFS, recurrence-free survival.

**Table 2:** Stratified analyses of the pooled HRs of overall survival with over-expressed BCAR4 in subgroup cancer patients.

Subgroups	Studies	HR (95% CI)	Significance (*P*-value)	Model	Heterogeneity *I^2^*, *P*-value
1 Tumor type					
1.1 BC	2	2.44 (1.22, 4.85)	0.011	Random	66.6%, 0.084
1.2 GI cancer	3	2.04 (1.55, 2.69)	0.003	Random	0%, 0. 810
1.3 Osteosarcoma	2	2.58 (1.38, 4.80)	<0.001	Random	0%, 0.627
1.4 Others	3	2.87 (1.66, 4.95)	<0.001	Random	0%, 0.863
2 Sample size					
2.1 <100	6	2.28 (1.78, 2.91)	<0.001	Fixed	0%, 0.693
2.2 ≥100	4	2.15 (1.56, 2.97)	<0.001	Fixed	0%, 0.521
3 Follow up (months)					
3.1 ≤60	6	2.42 (1.82, 3.22)	<0.001	Fixed	0%, 0.597
3.2 >60	4	2.08 (1.60, 2.71)	<0.001	Fixed	0%, 0.767
4 Survival analysis method					
4.1 Univariate	5	2.10 (1.67, 2.65)	<0.001	Fixed	0%, 0.463
4.2 Multivariate	5	2.57 (1.80, 3.66)	<0.001	Fixed	0%, 0.920

Abbreviations: BC, breast cancer; HR, hazard ratio; GI, gastrointestinal.

**Table 3:** Analysis of the pooled ORs of other clinicopathological features with over-expressed BCAR4 in cancer patients.

Outcome	No. of Studies	No. of Participants	OR (95% CI)	*P* value	Model	Heterogeneity Chi², *P*-value, *I²*
Age	5	318	1.38 (0.87, 2.20)	0.18	Fixed	5.27, 0.26, 24%
Gender	6	466	1.01 (0.69, 1.47)	0.96	Fixed	2.01, 0.85, 0%
Clinical stage	7	610	3.28 (2.33, 4.60)	<0.00001	Fixed	4.21, 0.66, 0%
Tumor size	2	228	1.26 (0.75, 2.12)	0.46	Random	3.62, 0.06, 72%
LNM	5	416	3.00 (1.95, 4.63)	<0.00001	Fixed	7.36, 0.12, 46%
DM	3	296	3.36 (1.88, 5.98)	<0.0001	Fixed	0.61, 0.74, 0%

Abbreviations: DM, distant metastasis; LNM, lymph node metastasis; OR, odds ratio.

## Abbreviations

BC: breast cancer
BCAR4: breast cancer anti-estrogen resistance 4
BRCA: breast invasive carcinoma
CI: confidence interval
CESC: cervical squamous cell carcinoma and endocervical adenocarcinoma
CNKI: China National Knowledge Infrastructure
CRC: colorectal cancer
DFS: disease-free survival
DM: distant metastasis
EMT: epithelial-mesenchymal transition
GC: gastric cancer
GEPIA: Gene Expression Profiling Interactive Analysis
GI: gastrointestinal
GLI2: glioma-associated oncogene 2
Hh: Hedgehog
HR: hazard ratio
K-M: Kaplan-Meier
lncRNA: Long non-coding RNA
LNM: lymph node metastasis
LUAD: lung adenocarcinoma
LUSC: lung squamous cell carcinoma
miRNAs: microRNAs
MFS: metastasis-free survival
N/A: not available
ncRNA: Non-coding RNA
NOS: Newcastle-Ottawa Scale
NSCLC: non-small cell lung cancer
OR: odds ratio
OS: overall survival
PC: Prostate cancer
PFS: progression-free survival
piRNA: piwi-interacting RNA
PRAD: prostate adenocarcinoma
RFS: recurrence-free survival
SARC: sarcoma
siRNA: short interfering RNA
STAD: stomach adenocarcinoma
TCGA: The Cancer Genome Atlas
YAP: Yes-associated protein
