# Supplementary material for: The Value of LncRNA BCAR4 as a Prognostic Biomarker on Clinical Outcomes in Human Cancers
Source: J Cancer. 2019 Oct 15;10(24):5992–6002. doi: 10.7150/jca.35113 (PMC6856575; doi:10.7150/jca.35113)

Figure S1. Scatter plots of BCAR4 expression levels between cancerous and normal tissues in patients with sarcoma (SARC), breast invasive carcinoma (BRCA), cervical squamous cell carcinoma and endocervical adenocarcinoma (CESC), colon adenocarcinoma (COAD), lung adenocarcinoma (LUAD), lung squamous cell carcinoma (LUSC), prostate adenocarcinoma (PRAD), rectum adenocarcinoma (READ), and stomach adenocarcinoma (STAD).

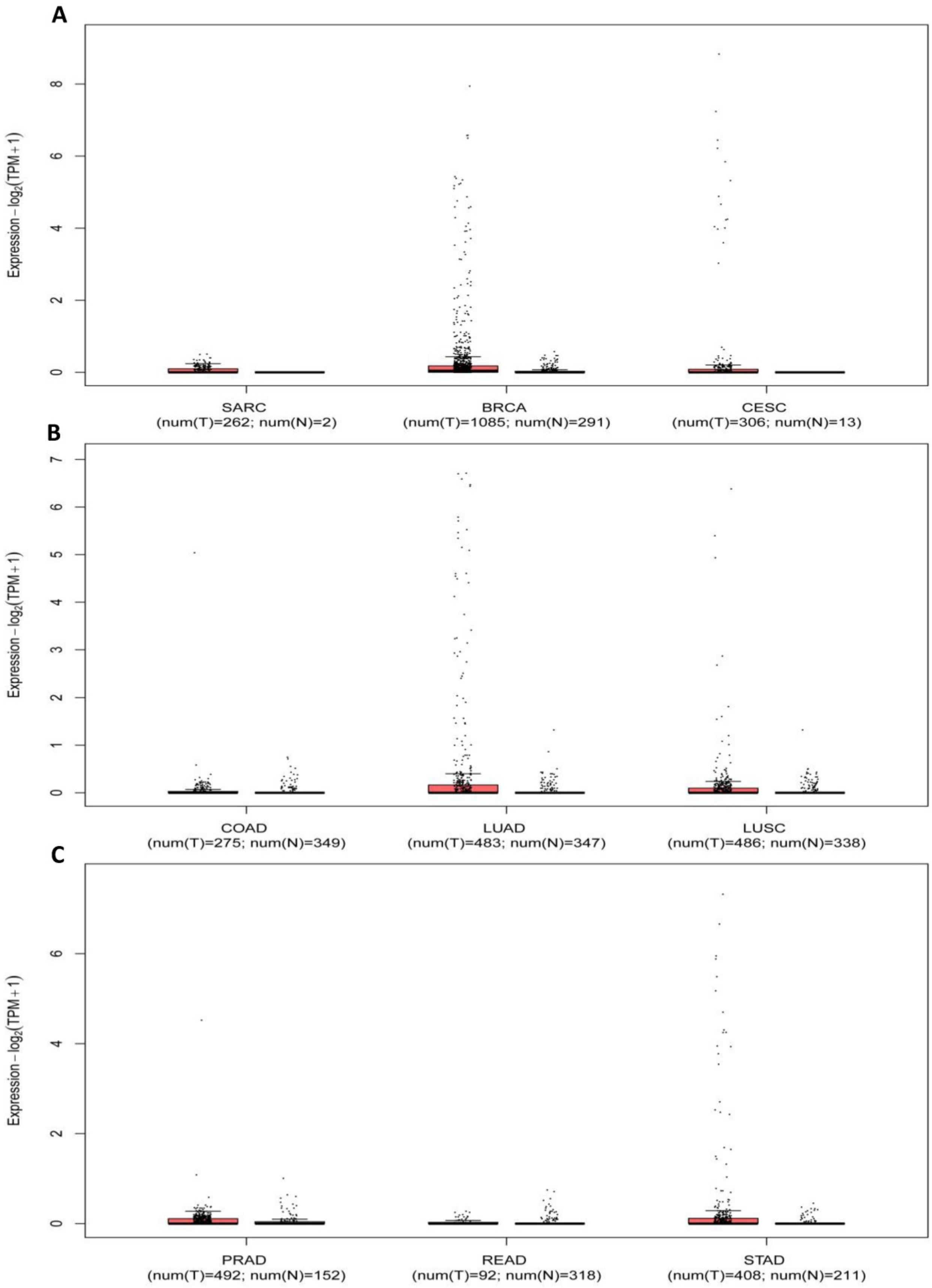

Supplement: Supplementary file 1 — Supplementary figures. [file jcav10p5992s1.pdf]
